# Supplementary figures and images for: Transcriptome analysis of genetically matched human induced pluripotent stem cells disomic or trisomic for chromosome 21
Source: PLoS One. 2018 Mar 27;13(3):e0194581. doi: 10.1371/journal.pone.0194581 (PMC5870938; doi:10.1371/journal.pone.0194581)

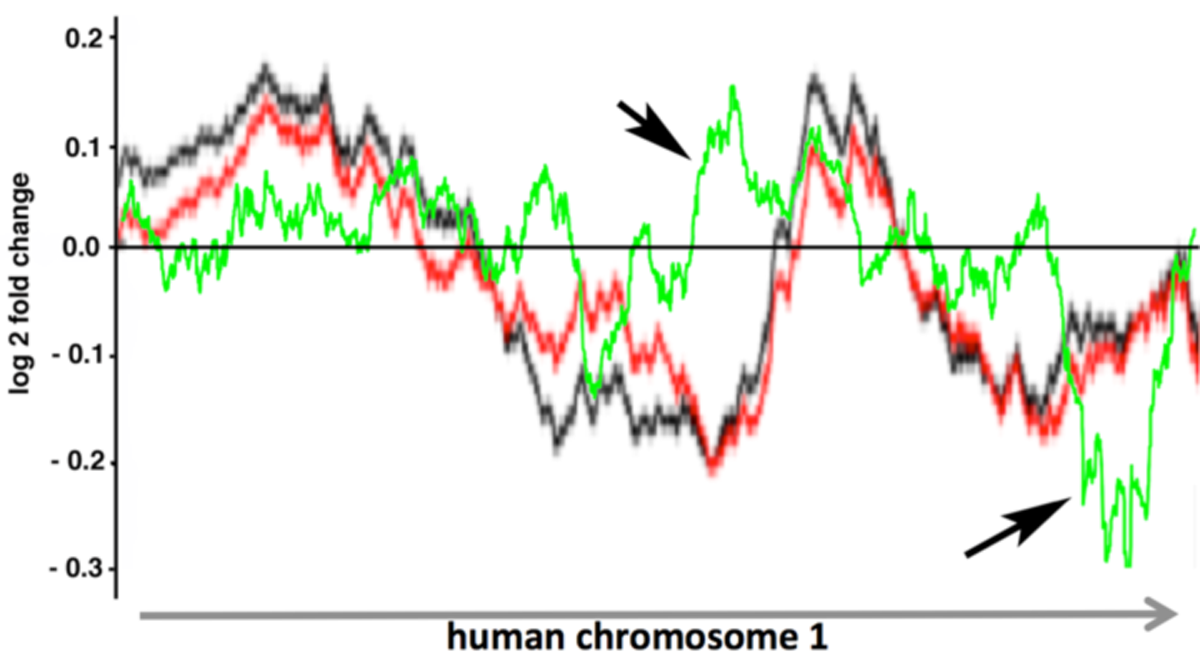

Supplement: S1 Fig — Lines show regions of increased or decreased gene expression in cells trisomic for chromosome 21 as calculated using the smoothing algorithm employed by Letourneau et al. The red (iPSCs) and black (fibroblast) traces are taken from the Letourneau study; the green trace is from the iPSCs used in this study. Note that there are significantly different expression domains calculated from the different studies (arrows). (TIF) [file pone.0194581.s001.tif]
